# Isla: integrating full-scale ISA semantics and axiomatic concurrency models (extended version)

**DOI:** 10.1007/s10703-023-00409-y

**Published:** 2023-05-12

**Authors:** Alasdair Armstrong, Brian Campbell, Ben Simner, Christopher Pulte, Peter Sewell

**Affiliations:** 1 Department of Computer Science, University of Cambridge, Cambridge, UK; 2 School of Informatics, University of Edinburgh, Edinburgh, UK

**Keywords:** Instruction set architecture, Axiomatic concurrency, Symbolic execution

## Abstract

Architecture specifications such as Armv8-A and RISC-V are the ultimate foundation for software verification and the correctness criteria for hardware verification. They should define the allowed sequential and relaxed-memory concurrency behaviour of programs, but hitherto there has been no integration of full-scale instruction-set architecture (ISA) semantics with axiomatic concurrency models, either in mathematics or in tools. These ISA semantics can be surprisingly large and intricate, e.g. 100k$+$ lines for Armv8-A. In this paper we present a tool, Isla, for computing the allowed behaviours of concurrent litmus tests with respect to full-scale ISA definitions, in the Sail language, and arbitrary axiomatic relaxed-memory concurrency models, in the Cat language. It is based on a generic symbolic engine for Sail ISA specifications. We equip the tool with a web interface to make it widely accessible, and illustrate and evaluate it for Armv8-A and RISC-V. The symbolic execution engine is valuable also for other verification tasks: it has been used in automated ISA test generation for the Arm Morello prototype architecture, extending Armv8-A with CHERI capabilities, and for Iris program-logic reasoning about binary code above the Armv8-A and RISC-V ISA specifications. By using full-scale and authoritative ISA semantics, Isla lets one evaluate litmus tests using arbitrary user instructions with high confidence. Moreover, because these ISA specifications give detailed and validated definitions of the sequential aspects of *systems* functionality, as used by hypervisors and operating systems, e.g. instruction fetch, exceptions, and address translation, our tool provides a basis for developing concurrency semantics for these. We demonstrate this for the Armv8-A instruction-fetch and virtual-memory models and examples of Simner et al.

## Introduction

A processor architecture specification should define, for any initial machine state, the set of all architecturally allowed observable executions—thus specifying the basic assumptions for programming and for software verification, and the correctness criterion for hardware verification.

Traditionally, industry architecture specifications have been large prose documents, sometimes with pseudocode descriptions of instruction behaviour. These prose specifications have often been combined with “golden” model simulators often written in C or C++. These specifications can be very large and complex—manuals for Arm and Intel number in the thousands of pages.

Architecture specifications have two main parts: a description of sequential instruction behaviour, and a memory model defining the relaxed-memory concurrent aspects of those instructions. Both of these aspects have been extensively studied in previous work. In those work, we focus primarily on two architectures: Armv8-A and RISC-V (with a particular focus on Armv8-A), although our approach could in principle be applied to any architecture such as $\times$86, POWER, or others.

For Armv8-A and RISC-V, there exist full-scale sequential models in Sail [1, 2], a domain-specific language for instruction-set architecture (ISA) specification, that are complete enough to boot real-world operating systems such as Linux. For Armv8-A this model is automatically derived from the authoritative Arm-internal specification [3], while for RISC-V it has been hand-written, and adopted by RISC-V International.

On the concurrency side, relaxed-memory semantics can be specified in two main styles: either as *abstract-microarchitectural operational* models, characterising observable behaviour with explicit out-of-order execution and buffering, or as *axiomatic* models, expressed as a predicate over complete candidate executions represented as graphs of memory events. For Armv8-A and RISC-V “user” concurrency, both exist [4–8], along with a “Promising Arm/RISC-V” variant [9]. For Armv8-A they have been proved equivalent [4, 10]; the authoritative vendor definition is the axiomatic one.

However, while an architecture *should* define the set of allowed executions for arbitrary programs, hitherto there has been no integration of full-scale ISA definitions with axiomatic concurrency models, either in mathematics or in tools (for operational models, this has only been done for RISC-V in RMEM [11]; other operational models have used small ISA fragments). Research and industry practice for relaxed memory semantics rely on making the semantics *executable as a test oracle*: not just a paper definition (in prose or mathematics), but tool-supported definitions that for small litmus test examples can *compute* the set of all allowed executions, that can then be compared against experimental data. Many tools have been developed for operational and axiomatic architectural concurrency models [7, 11–25], with axiomatic tools notably including the Herd tool of Alglave and Maranget [7, 20, 21] that can evaluate litmus tests w.r.t. axiomatic memory models specified in a relational-algebra style in the Cat language [26]. However, all of these previous tools for axiomatic models have (at best) used hard-coded ISA semantics that cover only small fragments of the complete architecture. For example, Zhang et al. [12] use an SMT solver-based approach for SoC verification, with a user-specified memory model (TSO or SC), however the “instruction level abstractions” they use are much more abstract than the ISA semantics we consider.

One particular challenge for the problem of integrating full-scale ISA specifications into an axiomatic concurrency setting is that the axiomatic models work as predicate over complete executions, and therefore cannot drive the ISA specification in a step-by-step manner, which is the approach taken for the RISC-V model in RMEM.

In this article we describe a tool, Isla, that integrates full-scale ISA specifications, in Sail, with arbitrary axiomatic models, in a Cat-derived language. We first build a generic symbolic execution library for Sail specifications. We use this to construct a tool for symbolically running binary litmus tests for any Sail ISA under any (non-recursive) axiomatic memory model, using an SMT solver. We equip it with a web interface to make it widely accessible, and illustrate and evaluate all this for Armv8-A and RISC-V. The symbolic execution engine is valuable also for other verification tasks: it has been used in automated ISA test generation for the Arm Morello prototype architecture, extending Armv8-A with CHERI capabilities [27], and for Iris program-logic reasoning about binary code above the Armv8-A and RISC-V ISA specifications with the Islaris tool [28]. Isla is available at https://isla-axiomatic.cl.cam.ac.uk for the online web interface and https://github.com/rems-project/isla for the source code and documentation.

Our approach has several key advantages, which all follow from the fact that mainstream industry ISAs are surprisingly large and intricate. The Armv8-A ISA specification is around 100k lines. It defines the sequential behaviour of the full instruction set in all its detail, including e.g. instruction decoding, behaviour at each exception level, register banking, floating-point, vector instructions, system registers, exceptions, address translation, virtualisation, security extensions, and a host of optional architectural features.

Simple litmus tests developed to investigate user concurrency have historically used only very few instructions and involved very little of this detail, and hand-written ISA models have sufficed, but even a ‘simple’ ADD instruction can, in reality, involve surprisingly much of the specification. If one wants to examine arbitrary compiler-generated code one needs many more instructions; and to develop systems concurrency semantics, e.g. covering the concurrency behaviour of instruction fetch, exceptions, or address translation, one might need any of the specification—and it would be exceedingly laborious and error-prone to reproduce it by hand in a hard-coded semantics.

By handling the full authoritative Armv8-A ISA, we automatically support litmus tests that use arbitrary instructions, and we enable research on systems concurrency, with high confidence that the instruction semantics follow the vendor specification. We demonstrate this by applying our tool to the model and examples for self-modifying code by Simner et al. [29], and extending our tool to support tests and models involving address translation and virtual memory by Simner et al. [30].

Our integration of these full Sail ISA specifications with axiomatic concurrency has identified several places where the ISA specifications needed modification to correctly give the intended behaviour in a concurrent setting, e.g. to remove or enforce additional ordering. Because this is based on authoritative Arm and RISC-V ISA specifications, the work should enable relaxed-memory behaviour to be included in the standard test-edit-debug cycle used in the development of such large and critical specifications.

This is an extended version of our CAV 2021 tool paper [31]. It contains additional background information on axiomatic concurrency and relaxed memory that was not included in the conference version, as well as detailing the upgrades to our tool since [31], primarily for systems litmus tests supporting address translation and virtual memory [30].

This article is structured as follows: In Sect. 2 we introduce more formally the concept of an axiomatic memory model, with the full version of the Armv8 axiomatic model that we use in this paper included as Appendix A. In Sect. 3 we describe the operation of our Isla tool for evaluating the behaviour of these memory models using symbolic execution of the Sail ISA models. Here we also describe how we generate the syntactic dependency information—crucial for linking the sequential specification with the concurrency model, but not well specified in either. In Sect. 4 we use instruction fetch and virtual memory as motivating examples, demonstrating how our tool supports us in building axiomatic concurrency models for these and other systems features. Section 5 contains a comparison of our tool with Herd, and some additional results from evaluating our tool on a large corpus of litmus tests.

## Axiomatic memory models

**Fig. 1:**
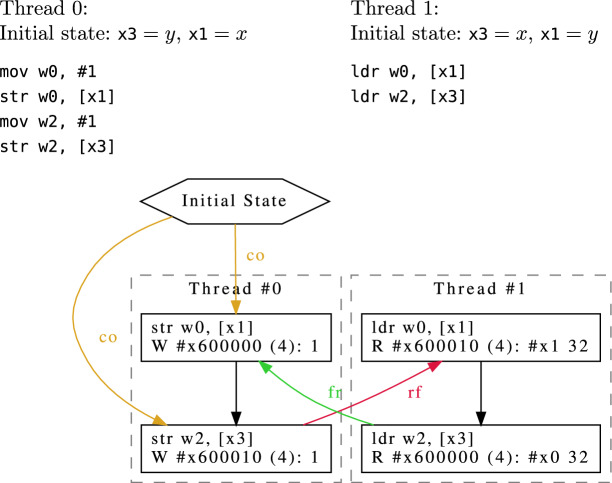
Message passing (MP) test for Armv8

Consider the small Arm program in Fig. 1. Thread 0 writes the value 1 to addresses *x* and *y*, in that order, while thread 1 loads *y* followed by *x*. In a sequentially consistent world, it would be impossible for thread 1 to observe $y = 1$ followed by $x = 0$, yet this can be observed on actual hardware with relaxed memory. Small programs of this kind that explore some aspect of the relaxed memory behaviour are called litmus tests. Note that the Arm assembly in Fig. 1, as well as subsequent assembly snippets in this paper, use the standard Arm convention that x0 and w0 refer to the same register, where w0 refers to the lower 32-bits of the register, and x0 refers to the full 64-bit width.

The graph in Fig. 1 shows this non-sequentially consistent allowed execution (where $x=\texttt {\#x}600000$ and $y=\texttt {\#x}600010$). The various labelled edges indicate relations between the initial state, and the load/store events. The unlabelled edges represent the program order in each thread. There are four relations of note in this graph:

- The program order (po), relating same-thread events in the order of the execution’s control-flow unfolding.
- The reads-from (rf) relation, relating write events to the read events that read from them. Note that in the graph we don’t draw these edges from the initial state.
- The coherence order (co) relation, a total order on memory writes corresponding to the sequence they propagate to memory
- The derived from-reads relation ($\texttt {fr = rf}^{\texttt {-1}}\texttt {;co}$), relating reads to same-address writes that are coherence-after the write they read from.

Note the cycle involving the reads-from (rf) relation, from-reads (fr) relation, and the program order. This kind of cycle would not be permitted under sequential consistency.

An *axiomatic memory model* is a predicate over such graphs. The Cat language [26] allows one to define such predicates using relations over the events in these graphs, and constraints over those relations, e.g. that specific relations are irreflexive, acyclic, or empty (or the negation of any of these). Relations are defined in a point-free relation-algebraic style, in terms of standard relational operators such as composition, intersection, and union.

## Implementation

Axiomatic relaxed-memory concurrency models, being expressed as logical constraints over candidate execution graphs, lend themselves to SMT solver-based tool implementations. For the instruction-semantics part of such a tool, the most direct approach would be to translate the ISA semantics (for the instructions that occur in a litmus test) directly into SMT and combine that with the axiomatic-model constraints, roughly along the lines of Alglave et al. [32]. That approach was followed by Simner et al. [29], which compiled Sail directly into SMT to test an axiomatic model for instruction-fetch tests, but using a small handwritten Arm fragment, rather than the full Sail model derived from the Arm-internal model. The problem with this direct approach is one of scale: as one covers more of the Arm semantics, the resulting SMT problem simply becomes too large to be practicable. For example, for a load instruction, the virtual address must be translated into a physical address, which is a complex process with a great deal of configurability—there may be zero, one, or two stages of address translation, the page size may vary, the number of levels used in the page table may differ, etc. This approach also required the top-level fetch-execute-decode loop to be handled specially, as one cannot translate such an unbounded loop directly into SMT, which imposes significant constraints on the shape of allowable tests.

In contrast, here we build and use a generic symbolic evaluation for Sail definitions using the Z3 SMT solver, which lets us compute the possible symbolic thread-local traces of each instruction, and hence of each thread (treating memory values as unknowns, left to the concurrency model constraints). It also lets us use the same fetch-decode-execute loop that is used for emulation and co-simulation (which embodies various architecture-specific subtleties).

### Symbolic execution for Sail

Sail is attractive for symbolic execution for several reasons. First, it is an intentionally simple language, lacking many of the features found in general-purpose languages. Second, it has to support very few programs, just the specifications of major ISAs, so (unlike tools for conventional programming languages) we can tune the execution to them. Third, almost all of the loops in these programs are bounded. Our starting point is the translation of Sail to C, for emulation [1]. This goes via a simple goto-language intermediate representation which is already well-suited for this task.

### Per-thread traces

For each litmus test thread this symbolic execution will produce a number of per-thread traces, each of which is a sequence of memory events (memory reads and writes, fences, register accesses, and so on) with the symbolic values of these events potentially being constrained by some SMT formula for the overall execution. Consider the Armv8-A instruction add x4, x3, #1. For this instruction, our symbolic evaluator generates (after some simplification of the generated SMT formula) an execution equivalent to just: 
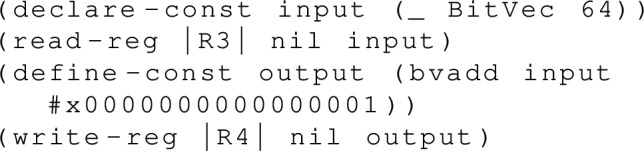
 where the SMTLIB formula is defined by the  and  statements, with  and  effects indicating which variables in the SMT formula correspond to the values read and written to registers (which are otherwise just treated as global variables) by the instruction. For more complex instructions, there are additional effects for memory accesses, cache maintenance events, barriers, and so on, as one would expect.

To give a sense for the complexity of the full Armv8-A ISA specification, we will show the Sail code from which this execution is derived. Instructions that touch memory are much more complex than this, e.g. with address translation potentially involving multiple page-table walks and many access checks. All that is also supported by our tool, and the additional features we have to support page tables and address translation are detailed in Sect. 4.2.

The main execute function for the add (and the related subtract) instruction reads the source register values, calls an auxiliary  function to compute the mathematical result, including new  flag values, and writes the target register value and (if the opcode requires it) those flag values. It handles subtraction by negating  and setting  before doing an addition. 
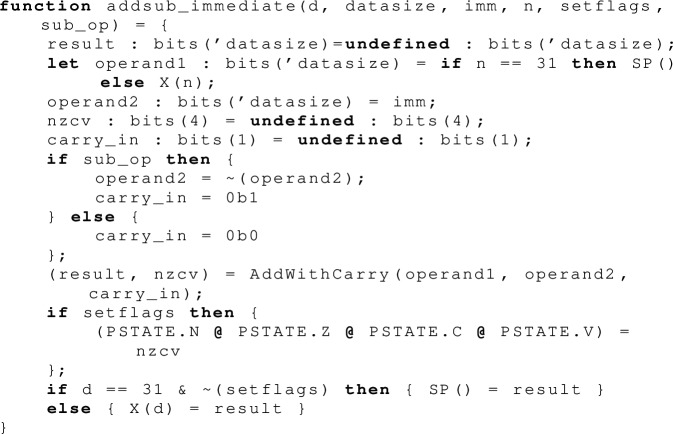


What look like register accesses in the above, e.g.  and , are actually indirected via register getter and setter functions, to handle the fact that in Armv8-A the stack pointer register  is *banked*: there is a different  register for each exception level. These functions therefore have to do another register read, not obvious from the opcode, of the register that holds the current exception level. 
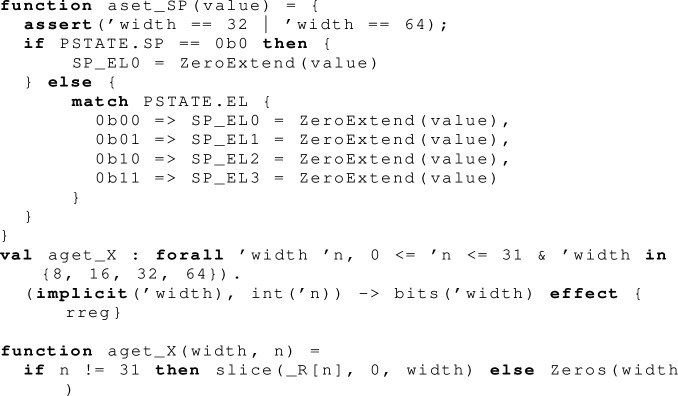


Finally we come to the actual (pure) arithmetic and computation of flag values, which is done over mathematical integers. This function computes both the signed and unsigned sum, which is used to determine if the carry and overflow flags need to be set. Note that our listing of the generated SMT problem earlier in this section did not include any mention of these flags, only interacting with the  and  registers. This is because a separate Armv8 instruction  (add with carry) is used when the flags are required.
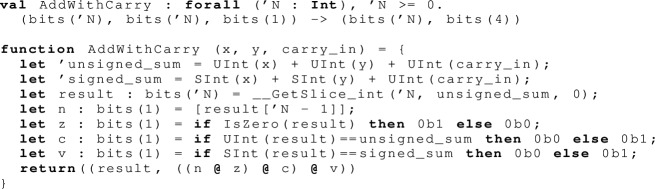


### Checking a litmus test

**Fig. 2:**
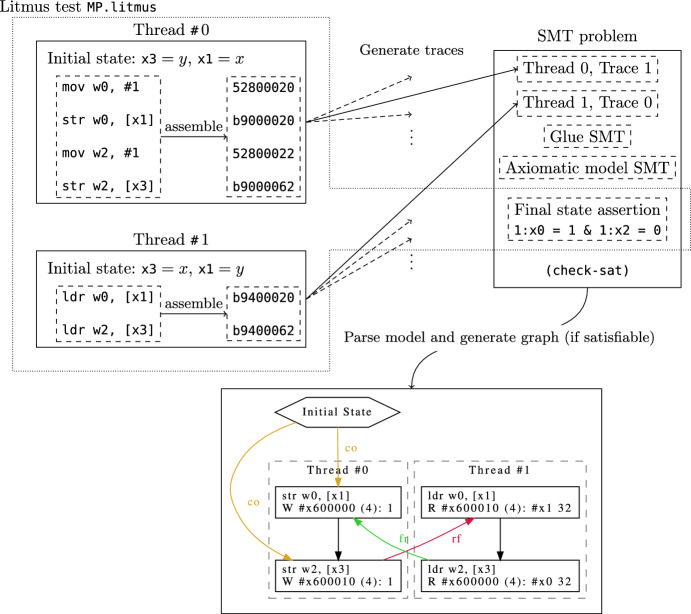
Overview of process for checking the allowed executions of a litmus test

Figure 2 shows the overall process of checking a litmus test. Tests can be supplied either in the .litmus format of previous axiomatic and operational tools [11, 21, 33], reusing the parser from Alglave and Maranget [21], or as a TOML file (a standard configuration file format, with libraries available for most languages). We first assemble the test with a conventional assembler into an ELF binary and load it into the representation of memory that will be used, before initialising the model with the program counter set to the entry point for each thread, then we symbolically execute the instructions in each thread separately, using the Sail semantics for each instruction, plus the same fetch-execute-decode loop in Sail we would use for emulation, to produce sets of per-thread traces as above. Treating litmus tests essentially as binaries, rather than the more-or-less ad hoc fragments of assembly abstract syntax used by earlier tools, accommodates the fact that the Armv8-A model does not define an abstract syntax, and reduces the gap between what the tool evaluates and what is run in experimental testing.

We then generate a candidate execution which is an SMT problem for every combination of the traces of each thread. This problem consists of the per-thread SMT formulae concatenated together (renaming variables as necessary to avoid name-clashes), combined with the axiomatic memory model (described in more detail below).

Finally, we need to generate some ‘glue’ SMT that connects the per-thread semantics with the memory model. For every effect in the per-thread SMT semantics we generate an enumeration of *events*, e.g. for an execution with two reads and two writes: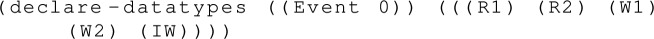 The event  is a special write event that represents the initial state. We generate relations such as  that relate events to their values as determined by the effects in the per-thread semantics, so if the second read event  read the value ,  would be true. We generate *syntactic dependency relations* for address, data, and control dependencies, discussed in detail in Sect. 3.4. Finally, each litmus test provides an assertion on the final state which specifies values expected in registers and memory after all threads have executed.

For the memory models, we define a straightforward translation from a subset of the Cat language which forbids recursion. The memory models we consider are all multi-copy-atomic, and all recursion in their definitions can trivially be replaced with (reflexive)-transitive closure. Herd’s let rec construct computes the least solution to a set of equations [26], which is tricky to represent in SMT, so we do not support it. We believe even relations such as Power’s (mutually recursive) preserved program order are nevertheless representable as SMT, so this limitation is mostly in our translation from Cat—instead of supporting let rec directly we currently allow the user to drop down to the level of plain SMTLIB definitions as needed to express such relations.

A satisfiable solution to the overall SMT problem described above thus represents an execution permitted by the architecture. Parsing the model generated by the SMT solver allows us to generate a graph of the execution by instantiating each relation in the model with the various events. If all generated SMT problems are unsatisfiable for every combination of per-thread traces then there are no permitted executions for the final assertion specified by the test. If desired we can repeatedly ask the SMT solver for additional distinct models until we have exhaustively explored all permitted executions.

### Syntactic dependency analysis

Axiomatic memory models for relaxed hardware architectures rely heavily on notions of address, data, and control dependencies between instructions. For example, consider the following assembly: 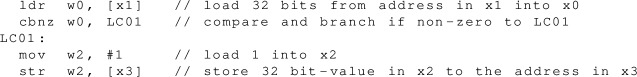 Here there is a control dependency between the load (ldr) and the store (str), as the value read by the load is used to determine whether the branch instruction  that precedes the store is taken or not. This control dependency exists regardless of whether the branch is taken or not—its existence is purely determined by the syntactic structure of the above code.

In general, existing ISA descriptions do not cover this aspect of the architecture well, as they are principally developed only to describe the sequential behaviour. Previous tools have either hand-coded dependency information, which is acceptable for cut-down ISA models but too laborious and error-prone at the scale of the ISA models we use, or used a heavyweight taint-tracking interpreter [2]. Our approach avoids both of these. It is similar to the latter, computing dependencies from the ISA specification, but building the footprint analysis atop our symbolic execution library requires only around 500 LoC.

To express dependencies, we need to associate each event in our candidate executions with the concrete assembly opcode/instruction that generated them, such as stlxr w0, x1, [sp] or ldaxr x2, [sp]. To do this we use a Sail function , called in each architecture’s fetch-decode-execute loop just after fetching an instruction; this adds a special effect to the candidate execution recording the instruction opcode. We also have another special effect that delimits each fetch-decode-execute cycle, so each effect such as  and  that would give rise to an event can be associated with an opcode, as well as its place in the total order defining the sequence of instructions that were executed by each thread. This lets us define a function $\textsc {instr} : E \rightarrow I$ which maps each event to the instruction that executed it.

In addition for each pair of events $e_1$ and $e_2$ in the same thread we have a function

$$\begin{aligned} \textsc {between} : E \times E \rightarrow [I] \end{aligned}$$

such that $\textsc {between}(e_1, e_2)$ returns the sequence of instructions that were executed between the instructions that executed $e_1$ and $e_2$. Note that due to loops and self-modifying code this isn’t always just the sequence of instructions between the two instructions executing $e_1$ and $e_2$ in the litmus test file.

For each instruction we also need to know its *footprint*: data about the instruction including which input registers it reads, which output registers it writes, whether it is a branch instruction, and so on. It also contains *taint* information—we need to know which registers writes may contain data ‘tainted’ by a memory read performed by a load, or which input registers ‘taint’ data written to memory. The Sail ISA specifications do not explicitly describe this footprint, so we are forced to derive it from the specification.

To do this we symbolically evaluate each opcode independently in a suitably unconstrained environment so as to capture all its possible behaviours. This can be computationally expensive due to the number of possible behaviours some instructions have, so we build a footprint cache to avoid re-computing this where possible. It turns out to be hard to distinguish ordinary branches from instructions that can cause an exception to occur, so we add a special branch address announce effect, created by a Sail function  that we add to branch instructions in the Sail specifications. This also enables the taint tracking for branch addresses we need for control dependencies. The taint tracking is achieved simply by looking at what sub-expressions in the generated SMT problem contain variables that also appear in the various effects in each trace. For example, if we see the following trace: 
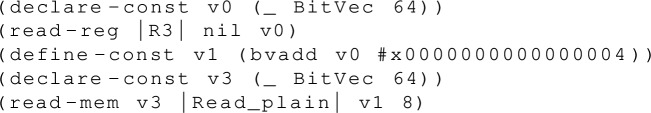
 We know that the register  is affecting the address of a subsequent memory read, as its value plus four is the address () of the subsequent  event.

From this analysis we generate four sets:

- The set of registers $R_i$ affected by data read from memory.
- The set of registers $W_i$ affecting data written to memory.
- The set of registers $A_i$ affecting the address of memory accesses.
- The set of registers $B_i$ affecting the address of any branch instruction.

Note that while we refer to registers here, in practice we apply this same method with slightly finer granularity by using smaller subfields of registers represented as structs in the Sail specification, e.g. PSTATE.N and PSTATE.C rather than the entire PSTATE register in Arm.

In addition we define for each instruction *i* a relation $F_i$ over registers which captures the possible register to register data-flow through each instruction. There are several choices for how we can compute $F_i$—firstly, we can simply say that $r_1F_ir_2$ if the instruction *i* reads register $r_1$ before writing $r_2$ in any trace of *i*. Second (and the method we currently use), is to be even more coarse-grained and say that $F_i$ relates all registers read by *i* to all registers written by *i*. In practice what we want is a method that is predictable and simple so that $F_i$ is obvious from the Sail source. It should also be mostly agnostic to the sequencing of Sail procedures within the ISA specification, as in the case of Armv8 we translate Sail from Arm’s ASL which wasn’t written with this kind of dependency analysis in mind, and we don’t want to radically re-sequence the code and risk introducing bugs in the sequential behaviour.

Using $F_i$, for any pair of events $e_1$ and $e_2$ such that

$$\begin{aligned} \textsc {between}(e_1, e_2) = i_0, \ldots , i_n \end{aligned}$$

we can the define a function

$$\begin{aligned} \textsc {flow}(e_1, e_2) = F_{i_0}; \ldots ; F_{i_n}. \end{aligned}$$

Now the address, data, and control dependency relations can be defined over any pair of memory events $e_1$ and $e_2$ as

- $\textsc {addr}(e_1, e_2)$ iff $[R_{\textsc {instr}(e_1)}];\textsc {flow}(e_1, e_2);[A_{\textsc {instr}(e_2)}]$ is non-empty
- $\textsc {data}(e_1, e_2)$ iff $[R_{\textsc {instr}(e_1)}];\textsc {flow}(e_1, e_2);[W_{\textsc {instr}(e_2)}]$ is non-empty
- $\textsc {ctrl}(e_1, e_2)$ iff there exists an *i* such that
  $$\begin{aligned}{}[R_{\textsc {instr}(e_1)}];\textsc {flow}(e_1, e_2);[B_i] \end{aligned}$$
  is non-empty and *i* is executed before $\textsc {instr}(e_2)$

Note that the $\textsc {addr}$, $\textsc {data}$, and $\textsc {ctrl}$ dependency relations we generate must be exact. If we under-approximate, we will allow executions that should be forbidden, and if we over-approximate we will forbid executions that should be allowed.

In some rare cases applying the above technique to the current Armv8 ISA specification does not result in the correct architecturally required dependencies due to $F_i$ over-approximating the register relationships, and our dependency analysis will therefore identify a dependency where there should not be one. To solve this we add some special Sail functions that give the specification author fine-grained control of the dependency calculation. For example, in Arm indirect branches we must ignore any dependency between the target register $\texttt {Xn}$ and the link register X30, even if it would otherwise appear to exist in our footprint calculation.

This is done by including a function in the Sail definition of indirect branches that tells the footprint analysis to ignore any relation it finds between the two registers, as shown: 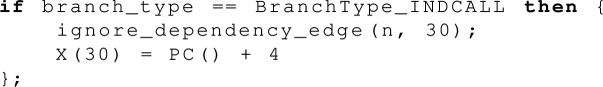 This inserts an annotation into the footprint execution trace which can be used by the footprint analysis when computing $F_i$ for the instruction—for all other purposes it is a no-op.

So far we have identified only two places in the Arm model where we need this more fine-grained control, in the aforementioned indirect branch instructions, and to ensure that there are no data-dependencies through the status register result of a store exclusive (strex) instruction.

In an ideal world this information should properly become part of the architecture specification, as mistakes in the dependency calculations could be a source of soundness bugs. The lack of support for this information in existing ISA specifications can partly be explained by the lack of tooling to properly explore the integration of ISA specifications with concurrency, something we hope a tool such as ours can address. In practice we imagine this would take the form of a 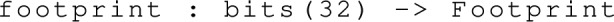 function, along side existing decode and execute functions, where  would be a data-type containing the various relations $R_i$, $W_i$, $A_i$, $B_i$, and $F_i$ we have described previously.

### Optimisations

Our symbolic execution always creates a new parallel task when we hit a non-deterministic branch in the Sail intermediate representation, and we do not merge these tasks at join points. This simplifies the symbolic execution engine significantly, and is a good strategy for litmus tests, as typically each litmus test will specify all its architectural state up-front (in terms of system registers). This means the amount of control-flow non-determinism for each thread is usually minimal. However, the Sail code of certain instructions can still cause unnecessary branching. To avoid this we have a static rewrite on our Sail intermediate representation that can take a function with if statements and rewrite it into a ‘linear’ form, e.g. as below:
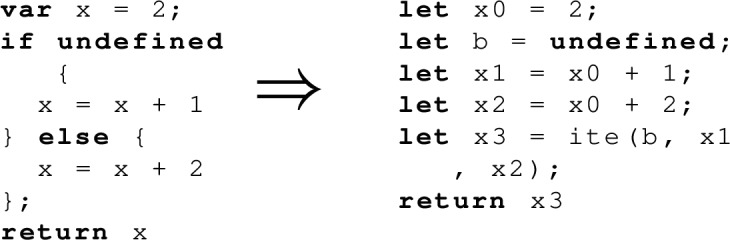


This works by translating the body of the function into single static assignment (SSA) form, then replacing the $\phi$-functions with if-then-else (ite) functions that translate into the SMT ite expression. This results in a more complex SMT expression, but less branching in the symbolic execution, so it is a trade-off, but often worthwhile. Since we are working with a limited set of known programs (our ISA specifications), the fact that we have to decide up front whether to apply this optimisation to any given function is not an issue, as it might be for a symbolic execution tool designed to work with any user-supplied program.

We can also apply this optimisation partially when required. For example, if the control flow graph contains nodes with side effects (such as accessing memory), then we must preserve the fact that some executions of the function may execute that node and have an observable side effect in the trace, whereas others will not. Here we can collapse the pure parts of the control flow graph as described above, leaving only control flow needed to handle the side effects. Provided there are no (externally visible) side effects this optimisation can merge any number of nested control flow structures into a single path.

An example of this would be the  function in the Sail Arm specification. This function takes several integer and enumeration arguments that describe a certain kind of fault that can occur during address translation, encoding this information into a bitvector that is returned (to subsequently be stored in a system register). Due to the amount of switching this function does on its various inputs to decide upon the correct bitvector encoding, this function has 33 paths through its control flow graph that the symbolic execution would need to explore. If we apply our partial linearisation optimisation however, we end up with just three. These correspond to the three behaviours the function may have: either it encodes the fault as a bitvector, the combination of arguments was invalid and an assertion fails, or it calls the  function indicating something that should never occur in the model.

### Web interface

**Fig. 3:**
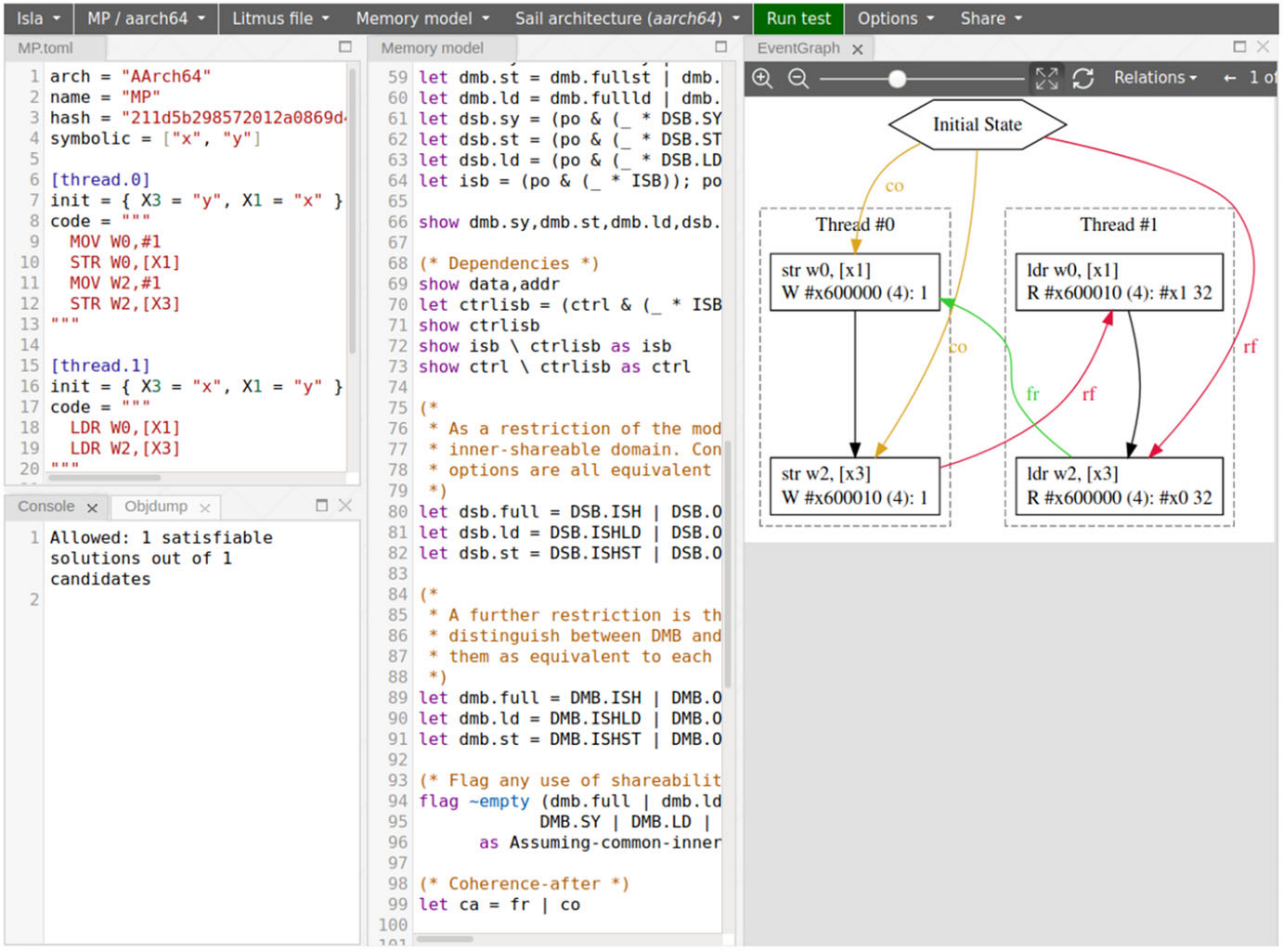
Web interface for the tool

Figure 3 shows the web interface we have developed for our tool, based on the web interface for the C memory model tool Cerberus-BMC by Lau et al. [34]. This can either be run locally, or via a website, https://isla-axiomatic.cl.cam.ac.uk.

## System litmus tests

As mentioned previously, one advantage of our tool is that because it supports full ISA specifications written in Sail, it enables easy experimentation with tests and models outside the scope of previous tools. In particular we are interested in *systems features* such as instruction fetch and data/instruction cache maintenance, and address translation. We call these systems features as they are primarily used by systems software such as operating systems and hypervisors.

Having tool support for these systems features is important for three main reasons:

1. It facilitates the development of axiomatic models by providing an automatic way to determine model behaviour on new litmus test programs as they are created.
2. It allows us to test that the more complex models involving systems features reduce to standard user-mode memory models when run on a large corpus of existing litmus tests.
3. It allows systems programmers to understand if the concurrency ordering required by their code is guaranteed to be preserved by the hardware

In this section we will give an overview of how our tool supports axiomatic concurrency modelling for such system features using instruction fetch and virtual memory as examples.

### Instruction fetch and cache maintenance

**Fig. 4:**
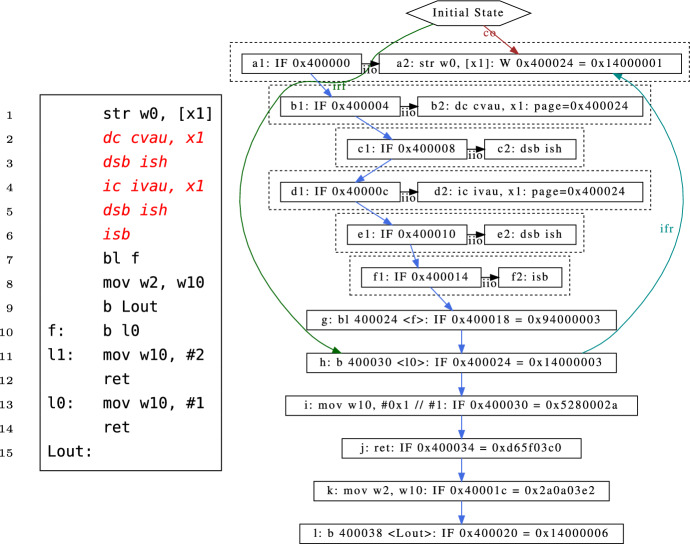
Self-modifying code litmus test SM+cachesync-isb

Simner et al. developed semantics for Arm instruction fetch and instruction/data cache maintenance [29]. Consider the litmus test in Fig. 4 [29, §3.3], a simple test involving self-modifying code. In order to run this test and the others in [29] our tool required only minimal changes: we had to add support for data-cache and instruction-cache maintenance events and relations for them in our Cat to SMT translation. Additionally we needed to generalise how we generated the rf (reads-from) relation to generate both the regular rf relation and the new irf (instruction-reads-from) relation. Because our tool already runs tests using a fetch-execute-decode loop, all the instruction fetch events were already available—we in fact filter them out when running user-mode tests.

In Fig. 4, the initial state register x1 contains the address of the label f, and register w0 contains the opcode for the branch instruction b l1. Without the highlighted cache-maintenance and barrier instructions on lines 2–6, the write of that opcode to f performed by the store on line 1 may or may not be observed before the instruction fetch for f, so at the end of the test the register w2 can contain either 1 or 2, depending on whether we branched to l1 or l0.

The highlighted instructions on lines 2–6 are a sequence of data-cache (dc) and instruction-cache (ic) maintenance instructions with requisite data and instruction barriers that must occur to guarantee that the write is observed by the instruction fetch, as documented by the Armv8-A architecture reference manual [5] and captured by the axiomatic model of Simner et al. [29]. The execution graph on the right of Fig. 4 shows this execution. This graph shows the fetch program order (fpo) relating all the instruction fetch events, while the instruction fetch analogues of reads-from (irf) and from-reads (ifr) as discussed in Appendix A are also shown.

When generating traces for a thread we normally do not assume anything about what other threads may be doing, but for self-modifying code this would clearly be problematic for performance, as it would imply that any other thread could modify any of this thread’s instructions arbitrarily. We therefore require the user to mark the memory locations that contain instructions that can be modified and provide in advance all the possible values they might take.

### Virtual memory and address translation

In this section we describe how Isla has been extended to support virtual memory litmus tests that interact with address translation and page tables. Semantics for virtual memory and address translation are important when considering the correctness of operating systems and hypervisors, as virtual memory is the primary mechanism by which process separation in operating systems (or guest separation in a hypervisor) is achieved. Describing a full model for address translation is beyond the scope of this paper, and we refer the reader to Simner et al. [30] which contains an extensive discussion of various models, and numerous tests demonstrating the envelope of allowed architectural behaviour.

Unlike instruction fetch, which required only minimal changes to our tool, supporting virtual memory is more of a challenge. To give an idea of the scope of the problem, we give a brief overview of how virtual memory and address translation works in a modern processor.

**Fig. 5:**
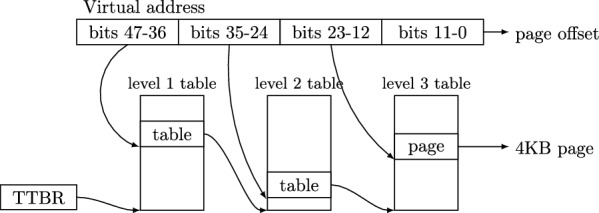
Address translation process

Figure 5 shows how a virtual address is translated into a physical address in Arm (and very similarly in most modern processor architectures). The virtual address is made up of indices into a series of translation tables, each containing a reference to the base address of next table in the sequence. The final table contains entries that point to concrete 4KB pages of memory. The address of the page and the final 12 bits of the virtual address which act as the offset in the page constitute the physical address. The base address of the first table is pointed to by a translation table base register (TTBR). These tables and the translation table base register are set up and managed by the operating system or hypervisor.

In practice the size and number of tables and the size of the page can vary depending on how the memory management unit (MMU) is configured. There are also various translation table base registers, which for example, may be used based on the exception level of the processor. Additionally Arm and other architectures support two stage translation, where the virtual address is first translated into an *intermediate physical address* and then into a physical address, repeating the process in Fig. 5 twice. This two stage translation process is used to implement virtualisation.

As one might imagine, it would be very expensive for a processor to actually perform each memory access to the translation tables every time a virtual address must be converted into a physical address, therefore the MMU has a cache of recent translations called the translation lookaside buffer (TLB). In addition to managing the translation tables, systems software such as operating systems and hypervisors must manually maintain this cache using TLB invalidate (TLBI) operations.

**Fig. 6:**
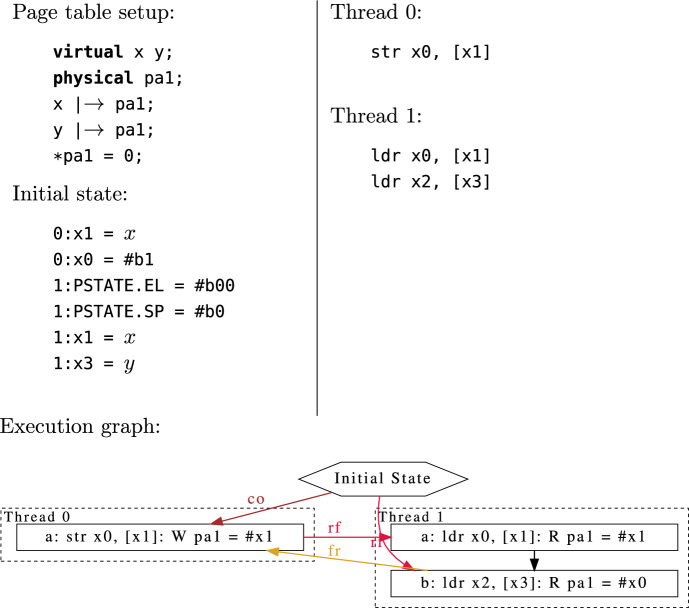
CoRR0.alias+po virtual memory test

Figure 6 shows an example of a very simple virtual memory litmus test, where we ask whether loads with different virtual addresses that map to the same physical address are allowed to be re-ordered if they read from different writes (the store in thread 0, and the initial state).

While the Arm (and RISC-V) ISA specifications contain code defining translation table walks, there is a problem we quickly encounter when trying to write litmus tests that interact with these features—we require a way to specify the initial configuration of the page tables in memory. In addition, the symbolic execution needs to be able to modify and walk over the table structure shown in Fig. 5.

The simplest possible solution might be to represent the initial page table state as a large binary blob loaded into memory before running each test. However we would still need a way of generating that blob, and all of its information would need to be encoded in the SMT solver, which would be impractical as the tables are many kilobytes in size. Instead, we have implemented a small language for describing page table configurations, as can be seen in Fig. 6. Here we declare two virtual addresses  and  and a physical address , such that both  and  are mapped to  using the *maps to* operator, . Finally we say that the memory location  starts the test as 0.

For most tests, we need more than a single concrete state for the page tables—imagine Fig. 5, except the arrows could point to different tables determined by some SMT formula. To support this our page table setup language describes a symbolic set of states the page tables could be in. For example: 
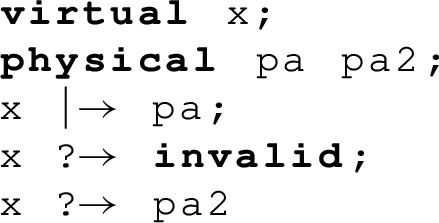


The  operator can be read as *maybe maps to*, and indicates that the translation in the above example could either return an invalid entry, , or . The single use of the  operator is important, as it provides the mapping that exists in the initial state, while any subsequent read could see any of the allowed values for the entry.

**Fig. 7:**
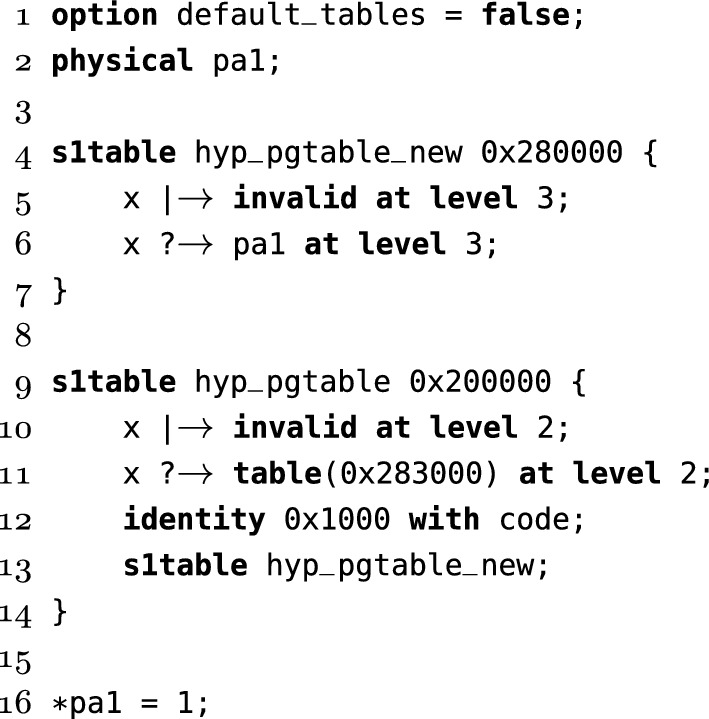
Page table setup for a hypervisor test

For more complex tests, we allow explicitly declaring tables, and creating mappings at specific levels, and with specific permissions. The  command creates a (stage 1) level 1 page table. Level 2 and 3 child tables are created implicitly as needed. For example in Fig. 7 we create an identity mapping in  at line 12 with code permissions, which is used for exception vectors. Nesting the  commands as seen on line 13 means that the addresses used for  are mapped in , allowing code to read and write the tables themselves. For simpler tests we have a default set of tables, but here this is disabled using the option on the first line.

In Isla, memory locations that are part of the page tables are initialised based on the provided description only when they are accessed, so only the parts of the page tables that are actually used are included in the candidate execution. To support this we have implemented generic machinery to support multiple *memory regions* in Isla, where each region can have a different programmable semantics, such as page table memory for virtual memory tests, or code memory for the ifetch tests. During symbolic execution Isla takes care to check that any single memory access is unambiguously within a single region.

#### Break before make

In Armv8 when modifying an existing mapping in the translation tables from a valid entry to another valid entry, the programmer must (in most cases) first *break* the mapping before writing the new entry. This break is done by first writing an invalid entry, using a  and a  to broadcast the write to all other threads and invalidate cached entries in any TLB. Only after this can the new mapping be created. This programming idiom is referred to as break-before-make (BBM).

Note however, that each of the writes in this BBM sequence may happen at different levels in the translation tables. Consider the translation in Fig. 5—we might invalidate it by writing an invalid entry to the level 1 table, before making it valid again by writing new entries at level 1 and 2. Detecting pairs of page table writes that violate this BBM property is not expressible in the Cat language as it requires encoding properties regarding the structure of the page tables. However, Isla allows us to express such a property as a predicate directly implemented in the SMTLIB language used by the solver. In [30] we write a predicate that can detect such BBM violations. This means we can determine allowable executions in two steps in the following way:

- Thread semantics + address translation memory model is satisfiable
- Thread semantics + address translation memory model + BBM predicate is unsatisfiable

If the BBM predicate is satisfiable when combined with the thread semantics and address translation, then the SMT solver can provide a model indicating exactly which pair of writes violated the BBM property.

## Results and comparisons

We evaluate our tool for correctness and performance with respect to Herd using previous corpora of tests.

We select 3798 litmus tests for both Armv8-A and RISC-V to compare between our tool and Herd—these tests include a representative set of features such as barriers and atomics, while exercising all of the basic litmus test shapes. All tests were run on a 2.6GHz Intel Xeon Gold 6240 CPU with 36 physical cores and 400GB of RAM. The tests are split into rough categories based on the contents of the tests. We ran 36 concurrent instances of both our tool and Herd across each set of tests, running Herd with the -speedcheck fast flag which causes it to stop enumerating executions when it resolves the final assertion in each test, which is the closest behaviour to how our tool behaves by default.

To assess correctness, we use a set of golden references for these above tests, for all of which the previous operational RMEM [11] and axiomatic Herd models and tools agree, and which have been extensively validated against hardware implementations. We confirm that our tool produces the same expected results as those tools for all the litmus tests, demonstrating the same set of possible behaviours for each test (when Isla is run in exhaustive mode).

To assess performance, Table 1 gives the total real execution time for each batch of tests.

**Table 1:** Isla and Herd performance across a set of litmus tests

Test set	Number of tests	Isla (s)	Herd (s)
Armv8-A basic 2-thread	1377	49.0	11.0
Armv8-A basic 3-thread	161	11.7	1.2
Armv8-A exclusives	23	20.2	1.5
Armv8-A DMB/LD	70	7.4	0.7
Armv8-A PPO	2020	209.3	16.2
RISC-V basic 2-thread	36	0.7	0.2
RISC-V AMOs	111	2.0	0.7

To assess performance, the Table 1 gives the total real execution time for each batch of tests. In general Herd is faster for nearly all tests, but this is not surprising given the amount of detail in the full-scale instruction semantics that we are using, particularly for Armv8-A. Our goal is not to be faster, but to support those full-scale ISA semantics while remaining fast enough for practical purposes. We achieve this: most tests take only a second or so to run, which is perfectly usable interactively. For example, given the Armv8-A basic 3-thread tests, for a single sequential run of the tests (not running any other tests in parallel), the shortest took 872 ms to run, while the longest took 1231 ms. The above batch times are similarly perfectly usable for (e.g.) regression testing while editing a model.

As for how the performance scales, the largest factor is the number of events in each candidate execution. As the number of events grows the performance becomes increasingly dominated by the SMT solver checking whether the final candidate execution is allowed, with the symbolic execution of each thread generating those candidates becoming negligible. As might be expected, the performance of the SMT solver can be quite unpredictable so it is hard to discern any particular patterns beyond the number of events.

We also evaluate our tool with respect to that of Simner et al. [29], for the instruction-fetch tests in Sect. 6 of their paper, which are currently not supported by Herd. Isla returns the expected results for all these tests, including the two tests (FOW and SM.F+ic) that were unsupported by the tool in that paper. In terms of performance, we note that the tool in [29] took 30 minutes to run just 90 of the 1377 basic 2-thread tests above, which is awkwardly slow for using in practice, whereas when limiting Isla to 8 cores (to more closely match the experimental setup in that paper) Isla tool will execute all 1377 in under 3 minutes. As we described in Sect. 3, this tool works by converting all the Sail source for the instructions directly into SMT, which was not practical even for the significantly cut down ISA model in [29].

We were additionally able to provide further validation that the Simner et al. model in Appendix A behaves as the standard Armv8-A model for non-self-modifying tests by showing that it behaves identically for all 3798 of the non-self-modifying tests above. The evaluation of Isla on a collection of virtual memory tests and models is described in [30].

## Conclusion and future work

In this article we have described our tool Isla for integrating full-scale ISA semantics in Sail and axiomatic concurrency models. We have shown that this integration allows us to explore systems features of architectures that have not been supported by prior tools.

There are several aspects that we plan to continue in future work: First, we aim to continue exploring interesting features of the Armv8-A (and upcoming Armv9-A) architecture. This includes features such as exceptions, as well as aspects of virtual memory and address translation that we have not yet considered.

Second, we have found that often we need information in our axiomatic models that is not easily expressible in the purely relational style of Cat. Ongoing work has involved extending our variant of the Cat language to support reasoning about arbitrary datatypes included by the Sail model in the events generated during symbolic execution. For example, in our virtual memory axiomatic model we have a primitive relation same-asid for TLB invalidates that share address space identifiers. We intend for such relations to be user definable purely within the memory model language without a need to add new primitives, in this case by using an address space identifier that is attached to the TLB invalidates by the Sail model.

Finally, we have found that as we add and combine additional system features, the number of events appearing in the candidate execution graphs grows significantly, sometimes up to hundreds of events. Adding optimisations that improve performance for these large execution graphs is something we intend to work on in the future.

## Figures and Tables

**Fig. 8:**
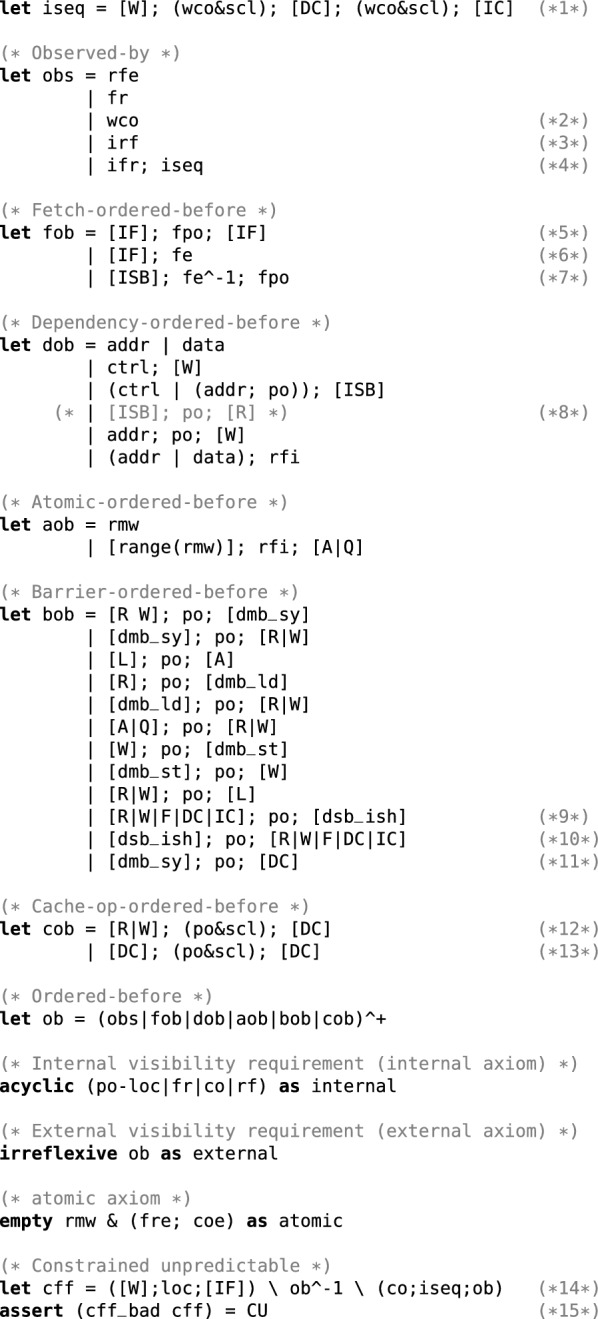
Arm-v8 Axiomatic model, as extended by [29]

## Data Availability

The datasets generated during and/or analysed during the current study are available from the corresponding author on reasonable request.

## A The Armv8-A axiomatic concurrency model

We recall the Armv8-A axiomatic concurrency model in the version we use here, which combines the official Arm specification for user concurrency (from the Armv8-A manual Issue B.a for Armv8.2-A, Arm DDI 0487B.a [5, 35]) with the additions for instruction fetch semantics by Simner et al. [29]. Figure 8 gives the full definition with a few minor presentational changes.

The model is expressed in terms of predicates on candidate executions, which are complete hypothetical executions of the input program, abstracted in terms of memory events and relations over them. The usual fundamental relations are program order (po), reads-from (rf), coherence (co), and from-reads (fr), as described in Sect. 2.

The Cat concrete syntax for relational algebra uses [X] for the identity on a set X, ; for composition,   for complement, ⌃-1 for relational inverse, | and & for union and intersection, and * for product. Additionally, we use ⌃+ and ⌃* for the transitive closure and reflexive-transitive closure respectively. By convention relations are suffixed e or i to indicate if they are restricted to their inter-thread (external) or intra-thread (internal) parts.

The model has four axioms. The internal axiom is a standard per-location-SC/coherence axiom. Roughly, this states that for each location the reads and writes to that location appear in some sequentially consistent order between all the threads. This forbids undesirable executions where threads would be able to read data written in the future and other such behaviour.

Second, the atomic axiom specifies the atomicity guarantees given by load/store exclusive pairs and atomic memory operations.

Third, the external axiom is the “main” axiom. It essentially requires that the ordering induced by the interaction across threads, captured by the obs (“observed by”) relation, is compatible with the thread-internal ordering fob|dob|aob|bob|cob. Here:

- fob is instruction-fetch related ordering,
- dob is ordering resulting from data and control dependencies,
- aob is ordering around exclusive instructions and atomic memory operations,
- bob is barrier ordering, and
- cob is ordering due to cache maintenance operations (such as DC and IC instruction).

Finally, the fourth axiom (15), related to instruction fetching, is explained below, alongside the other additions for instruction fetch and cache maintenance semantics where this model differs from [5]:

- The candidate execution has the following data:
  - events for instruction fetches (IF) as well as instruction and data cache maintenance operations (IC and DC);
  - the $\texttt {CU}$ bit, indicating constrained unpredictable executions; and
  - the relations irf, relating a write with instruction fetches that read from it; wco, extending the co coherence relation to include cache maintenance operations; fpo, the program order relation between instruction fetch events; fe, relating instruction fetches with any event originating from the execution of the fetched instruction; and scl, relating same-cache-line events.
- obs includes the extended coherence order wco (2), and orders any instruction fetch to be after the write it read from (3) and before any from-reads-related write that is sufficiently synchronised (4 and 1);
- fob orders fetches in fetch-program-order, (5), fetches before the instruction’s execute event (6), and instruction fetches after program-order earlier ISBs (7);
- [ISB]; po; [R] (8) in dob is subsumed by the orderings in fob, so is commented out here.
- bob contains ordering created by dsb_ish (9 and 10) and ordering of DC instructions with respect to dmb_sy barriers (11);
- the model defines cff, the could-fetch-from relation, that for a given instruction fetch captures the writes the fetch could have read from, including the one it did read from (14); and
- asserts that certain executions have constrained unpredictable behaviour (15): if this set contains more than one write and if one of these is the write of an instruction that is considered to be not concurrently modifiable:

$$\begin{aligned} \texttt {cff\_bad}\ \texttt {cff} = \exists i \in \texttt {IF}. \;&| \{ w | (w,i) \in \texttt {cff} \}| > 1 \; \wedge \\&\exists w. (w,i) \in \texttt {cff} \wedge \lnot \textsf {concurrently-modifiable} \ (\textsf {val} \ w). \end{aligned}$$
